# Early life stress exacerbates behavioural and neuronal alterations in adolescent male mice lacking methyl-CpG binding protein 2 (Mecp2)

**DOI:** 10.3389/fnbeh.2022.974692

**Published:** 2022-08-23

**Authors:** Jose Vicente Torres-Pérez, Elena Martínez-Rodríguez, Anabel Forte, Carlos Blanco-Gómez, Oliver Stork, Enrique Lanuza, Mónica Santos, Carmen Agustín-Pavón

**Affiliations:** ^1^Unitat Mixta d’Investigació en Neuroanatomia Funcional, Departament de Biologia Cel⋅lular, Biologia Funcional i Antropologia Física, Universitat de València, València, Spain; ^2^Department of Statistics and Operational Research, Universitat de València, Valencia, Spain; ^3^Department of Genetics and Molecular Neurobiology, Institute of Biology, Otto-von-Guericke University, Magdeburg, Germany; ^4^Center for Neuroscience and Cell Biology (CNC), University of Coimbra, Coimbra, Portugal; ^5^Institute for Interdisciplinary Research, University of Coimbra (IIIUC), Coimbra, Portugal

**Keywords:** c-FOS, doublecortin, maternal separation, reelin, Rett sydrome

## Abstract

The methyl-CpG binding protein 2 gene (*MECP2*) encodes an epigenetic transcriptional regulator implicated in neuronal plasticity. Loss-of-function mutations in this gene are the primary cause of Rett syndrome and, to a lesser degree, of other neurodevelopmental disorders. Recently, we demonstrated that both *Mecp2* haploinsuficiency and mild early life stress decrease anxiety-like behaviours and neuronal activation in brain areas controlling these responses in adolescent female mice. Here, we extend this work to males by using *Mecp2*-null and wild type adolescent mice subjected to maternal separation and their non-stressed controls. We assessed their behavioural responses in a battery of anxiety-provoking tests. Upon exposure to an elevated plus maze in aversive conditions, we evaluated changes in c-FOS expression in stress- and anxiety-related brain regions. In addition, we assessed the impact of maternal separation in neuronal maturation using doublecortin and reelin as surrogate markers. Mutant males showed reduced motor abilities, increased activation of the olfactory bulbs, probably due to breathing abnormalities, and decreased activation of the paraventricular thalamic nucleus, when compared to wild type mice. In addition, maternal separation increased the number of immature doublecortin-like neurons found in *Mecp2*-null animals. Moreover, this work shows for the first time that reelin is decreased in the mutant animals at the olfactory tubercle, piriform cortex and hippocampal dentate gyrus, an effect also associated to maternal separation. Taken together, our results suggest that maternal separation exacerbates some phenotypical alterations associated with lack of MeCP2 in adolescent males.

## Introduction

Loss-of-function mutations in the methyl-CpG binding protein 2 gene (*MECP2*), located in chromosome Xq28 and encoding the methyl-CpG binding protein 2 (MeCP2), are the major cause of Rett syndrome (RTT). In its classical presentation, RTT patients show normal development followed by a post-natal decline that is characterised by motor impairments, breathing abnormalities, loss of speech, intellectual disability, repetitive behaviours, and autistic-like features among others (Amir et al., 1999). In addition, anxiety is a prominent component in RTT (Barnes et al., 2015).

Mutations in the *MECP2* gene are considered lethal in hemizygosis and thus RTT has been regarded as a neurodevelopmental disorder affecting mostly females. Nonetheless, *MECP2* mutations have also been identified in male patients with intellectual disabilities (Orrico et al., 2000; Kudo et al., 2002), as well as cases of somatic mosaicism associated with RTT (Topçu et al., 2002; Venâncio et al., 2007). Additionally, *MECP2* duplication syndrome (MDS), which only manifests in males, is characterised by intellectual disability, heightened anxiety-like behaviours, seizures, and recurrent respiratory infections among others (D’Mello, 2021; Pascual-Alonso et al., 2021). The plethora of *MECP2*-related disorders demonstrates that this gene occupies a central role in the post-natal development of the brain in both sexes.

The levels of MeCP2 protein in the central nervous system are low during embryogenesis. However, its expression increases during post-natal stages, where it plays a critical role for neuronal maturation and synaptic pruning (Johnston et al., 2001). MeCP2 levels are high in mature neurons, where it seems to be involved in processes of activity-dependent regulation of gene expression (Lee et al., 2014, 2021).

MeCP2 is a multidomain epigenetic remodelling protein that acts in a context-dependent manner to silence and/or promote transcription (Sharifi and Yasui, 2021). Expectedly, MeCP2 is suggested to play a key role during post-natal “critical periods,” where the developing nervous system is in a pose state of high sensitivity to certain environmental factors.

MeCP2-dosage seems to have an impact on the neuronal differential epigenetic imprinting in response to the presence/absence of certain environmental inputs, which might result in altered behavioural manifestations later in life. Giving support to this, we have recently demonstrated that early life stress (ELS) in the form of maternal separation (MS) influences anxious behaviours, a prominent component of RTT’s behavioural phenotype (Barnes et al., 2015), in a female murine model of RTT (Abellán-Álvaro et al., 2021). Particularly, we demonstrated that, although adolescent *Mecp2*-heterozygous (*Mecp2*-het) females showed a phenotype of reduced anxiety-like behaviour during basal conditions, maternal separation (MS) was able to further reduce anxiety-like behaviours in both *Mecp2*-het and their wild-type (WT) littermates in the elevated plus maze (EPM). Those behavioural changes correlated with decreased neuronal activation at the hypothalamic paraventricular nucleus (Pa), thus supporting the role of MeCP2 in the reprogramming of the hypothalamic-pituitary-adrenal (HPA) axis and the differential response to anxiogenic scenarios during adolescence.

Here, we aimed to explore how the interaction between *Mecp2* deficiency and ELS shape behavioural and cellular phenotypes known to be affected by those variables on male mice. Thus, we investigated anxiety-like and depression-like behaviours and their neural activation correlated by means of c-FOS. We report the data derived from sibling males from the same Mecp2-het study. Thus, animals from corresponding genotypes (*Mecp2*-null vs. WT) were exposed to the ELS’s model of MS. At adolescent stages, animals were subjected to a battery of behavioural tests to assess anxiety-like [EPM and open field (OF)] and depression-like [forced swimming test (FST)] behaviours. After a second exposure to the EPM (in bright, aversive conditions), animals were sacrificed, and FOS proto-oncogene (c-FOS) immunohistochemistry was used to analyse neuronal activation in brain areas previously implicated in anxiety- and stress-related functions. Additionally, here we sought to assess the impact of the ELS on neuronal maturation in those male mice, so we also analysed the expression patterns of doublecortin (DCX) and reelin in the relevant neurogenic areas.

## Materials and methods

### Animals

For this study, we used *Mecp2*-null males and their WT littermates. We purchased *Mecp2*-het females from Jackson Laboratory [stock #003890, B6.129P2(C)-Mecp2^TM 1.1Bird/J^] and maintained breeding pairs, by crossing these females with C57Bl/6J WT males. Experimental animals were weaned at postnatal day (PND) 23 and housed in groups of 2–5 animals in standard laboratory cages with controlled humidity and temperature (22°C), a 12:12-h light/dark cycle, and water and food available *ad libitum*. Genotyping was performed with DNA extracted from ear biopsies according to the corresponding protocol supplied by Jackson Laboratory. The males used in this study were siblings of the females used in a recently published paper (Abellán-Álvaro et al., 2021). Body weight of the animals was measured before starting the behavioural experiments.

All the procedures were carried out in strict accordance with the EU directive 2010/63/EU. The protocols were approved by the Ethics in Animal Experimentation Committee of the University of Valencia.

### Early life stress and study design

**FIGURE 1:**
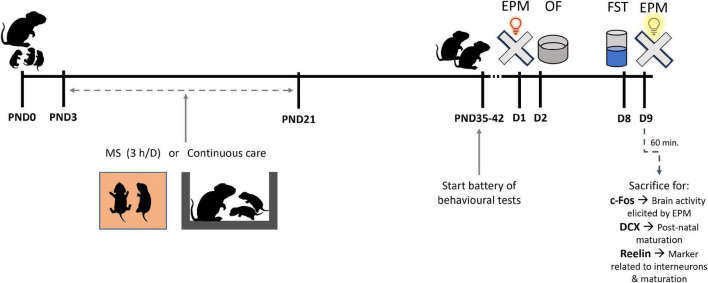
Diagram of the experimental design. In the litters assigned to maternal separation (MS), pups were separated from the dam for 3 h per day between postnatal days (PND) 3 and 21. After MS, pups were returned to their home cage. Behavioural testing started at 5 weeks of age with at least 24 h gap between tests consisting on: elevated plus maze (EPM), open field (OF), forced swim test (FST). The day after FST, animals were subjected to a new EPM but in bright light (more aversive) and 1 h after this test animals were transcardially perfused to obtain brain samples for histological experiments.

To induce ELS we used the MS protocol. From PND3 to PND21 pups (WT-MS, *n* = 15; *Mecp2*-null-MS, *n* = 18) were separated from the dam as a group, and kept in a new cage filled with sawdust and warming red light for 3 h per day during their dark phase (between 9 am and 5 pm as they had inverted cycle), after which pups were returned to their home cage. Control animals (WT-naive, *n* = 15; *Mecp2*-null-naive, *n* = 12) were maintained undisturbed with their dams in the home cage until weaning. Behavioural testing was performed starting at 5 weeks of age during the dark phase of the animals. Three of the animals in the *Mecp2*-null-MS and three of the animals in the *Mecp2*-null-naïve groups died after the first behavioural test, and could not complete the battery. Premature death of the *Mecp2*-null animals, together with earlier symptoms, is a common and expected feature of this murine model (Guy et al., 2001; Ribeiro and MacDonald, 2020). Hence, these animals were excluded from the analysis and the final numbers of *Mecp2*-null-MS and *Mecp2*-null-naive groups were *n* = 15 and *n* = 9, respectively. The behaviour of mice was tracked using an automated tracking system (ANY-maze™ Video Tracking System, Stoelting Co.). To ensure there was no interference between behavioural tests, the order of testing was from less aversive (EPM, OF) to more aversive (FST, EPM in bright light). Additionally, animals were allowed to recover for a minimum of 24 h between tests (Figure 1). An hour after last behavioural assay, a subset of animals was perfused (see below) for histological analysis.

### Elevated plus maze

Mice were tested in the EPM in two sessions. The first EPM session was the first test of the behavioural battery, under red light conditions. The second EPM session was the last test of the battery and was performed under bright light, a more aversive condition. The maze consisted of two opposing closed arms (in cm, 30 length × 6 wide × 15 height) and two opposing open arms (in cm, 30 length × 6 wide) extending from a common central region (6 cm × 6 cm) to form a “plus” shape. The arms were elevated 40 cm above the floor. In a single 5-min trial, animals were placed in the centre of the maze and the total distance travelled, and time spent in the open and closed arms were recorded. If differences in total distance travelled were reported, the number of entries into the open arms where normalised against this parameter (OAE/distance).

### Open field

Animals were allowed to explore an open field arena (50 cm × 50 cm × 50 cm) in a 20-min session, under red light illumination. The total distance travelled and time immobile were used as measures of activity and the percentage of time spent in a predefined centre area (25 cm × 25 cm) *versus* the rest of the arena was used to assess anxiety-like behaviour.

### Forced swimming test

This test has been traditionally used to test depressive-like state in rodents, although it might also evaluate behavioural reactivity to a stressful situation (Armario, 2021). Mice were put in an inescapable transparent jar filled two thirds of water (2 L beaker), and allowed to swim for a 5-min period. The latency to first immobility time and the time animals spent immobile were measured. Mice were carefully dried out with paper towels before they were returned to their home cage.

### Histology

A random subset of the animals used in the behavioural battery was selected for anatomical studies. Animals (WT-naïve, *n* = 4; *Mecp2*-null-naïve, *n* = 4; WT-MS, *n* = 7; *Mecp2*-null-MS, *n* = 5) were deeply anaesthetized 1 h after the last EPM session (bright conditions), using a mixture of ketamine (75 mg/kg) and medetomidine (1 mg/kg) and transcardially perfused with saline solution followed by 4% paraformaldehyde in 0.1M phosphate buffer (PB, pH 7.4). Brains were carefully removed from the skull, post-fixed in the same fixative for 4 h and placed into a 30% sucrose solution in 0.1M PB saline (PBS, pH 7.6) until they sank. The brains were then frozen and cut in six parallel series of 40-μm-thick coronal sections with a freezing microtome. Free-floating sections were frozen in 30% sucrose in PB/0.02% sodium azide (0.1 M pH 7.4) for their posterior processing.

### Immunohistochemistry

#### c-FOS and reelin immunohistochemistry

Two out of the six brain series of each subject were processed for the immunohistochemical detection of either c-FOS or reelin. To do so, endogenous peroxidase was quenched with 1% H_2_O_2_ in tris-buffered saline (TBS) (0.05M, pH 7.6) for 30 min at room temperature (RT). Next, sections were incubated in blocking solution containing 3% normal goat serum (NGS) in TBS-triton X100 (Tx) 0.3% for 1 h at RT. Sections were incubated in rabbit anti-c-FOS antibody (1:10000, sc-52, Santa Cruz Biotechnology, Santa Cruz, CA, United States) or mouse anti-Reelin antibody (1:1000, MAB5364, EMD Millipore) in TBS-Tx with 3% normal goat serum (NGS) for 24 h at 4°C, followed by an incubation with biotinylated goat anti-rabbit IgG (1:200, BA1000, Vector Laboratories, Peterborough, United Kingdom) or biotinylated goat anti-mouse IgG (1:200, BA9200, Vector Laboratories, Peterborough, United Kingdom), respectively, in TBS-Tx with 2% NGS for 2 h at RT. Afterward, sections were incubated in ABC Elite (1:50, PK-6100, Vector Laboratories) in TBS-Tx for 90 min at RT. Finally, the resulting peroxidase labelling was revealed with 0.025% 3-3’-diaminobenzidine (DAB, Sigma, St. Louis, MO, United States) in PB (0.1 M, pH 8.0) and 0.01% H_2_O_2_ for 25 min. Sections were mounted onto gelatinized slides, dehydrated in alcohols, cleared with xylene and cover-slipped with Entellan (Merck Millipore, Burlington, MA, United States).

### Doublecortin immunofluorescence

We obtained a simultaneous labelling of DCX and NeuN (used to delimitate areas of interest) in one out of the six parallel series. Briefly, brain slices were incubated with 1% sodium borohydride (NaBH_4_) in 0.05M TBS for 30 min at RT to eliminate the endogenous fluorescence of the tissue. Next, non-specific binding was blocked by incubating slices in 4% Normal Donkey Serum (NDS) in 0.05 TBS and 0.3% Triton-X100 for 1 h at RT. Subsequently, sections were incubated for two nights at 4°C with a mix of primary antibodies: mouse anti-NeuN IgG (Chemicon, MAB377) diluted 1: 2,500 and goat anti-DCX IgG (Santa Cruz Biotechnology, Inc., sc-8066) diluted 1: 250, in 0.05M TBS pH 7.6 with 2% NDS and 0.3% Triton-X100. Afterward, brain slices were incubated for 90 min at RT in darkness, with a mix of the two secondary antibodies, Alexa Fluor 488-conjugated donkey anti-mouse IgG (Invitrogen, A21202) and Alexa Fluor 568-conjugated donkey anti-goat IgG (Jackson Immunoresearch, 705-025-147), both diluted 1:250 in 0.05M TBS pH 7.6 with 2% NDS and 0.3% Triton-X100. Sections were thoroughly rinsed between each step in TBS. Finally, sections were mounted in slides with 0.2% gelatine in TB, and cover-slipped with FluorSave TM Reagent.

### Image acquisition, processing, and mapping

After c-FOS immunohistochemistry, we quantified c-FOS-positive nuclei in representative levels according to the stereotaxic atlas (Paxinos and Franklin, 2004) of nuclei relevant for cognitive, exploratory, hyperventilation and/or stress- and anxiety-driven functions, as follows: dentate gyrus of the hippocampus (DG; Bregma –1.34/–2.92 mm, 2 sections per animal), olfactory bulb (OB; Bregma 3.20/2.58 mm, 2 sections per animal), paraventricular hypothalamic nucleus (Pa; Bregma –0.7/–1.06 mm, 2 sections per animal) (Abellán-Álvaro et al., 2021), paraventricular thalamic nucleus (PV; Bregma –0.94/–2.18 mm, 4 sections per animal) (Kirouac, 2015), piriform cortex (Pir) layer 2 (Bregma 1/–2 mm, 4 sections per animal) (Martínez-García et al., 2012) and ventral part of the lateral septum (LSV; Bregma 0.62/–0.10 mm, 2 sections per animal) (Abellán-Álvaro et al., 2021). Photomicrographs of these frames were obtained in both hemispheres (when possible) using a digital camera (Leica DFC495, Wetzlar, Germany) attached to a microscope Leitz DMRB (Leica AG, Wetzlar, Germany) with a 10–20× objective, depending on the area (Counting frame of 881 × 661 or 441 × 330 μm). c-FOS-immunoreactive nuclei of each photomicrograph were counted using Fiji (Schindelin et al., 2012) by an experimenter blind to the genotype or group of the animals. Images were converted to grey scale, normalised to expand the histogram, binarised by Phasalkar’s method (Phansalkar et al., 2011) and remaining particles quantified as positive nuclei.

We analysed DCX-ir with a microscope equipped with both conventional light and fluorescence lamps (Leica Leitz DMRB), equipped with a digital camera (Leica DFC495) and software LAS v4.3 at *a priori* selected Bregma levels, according to stereotaxic coordinates (Paxinos and Franklin, 2004). A researcher unaware of the condition of the animals recorded the number of DCX-ir cells using the Multipoint plugin of the Image-J image analysis software (NIH), by classifying them in tangled or complex (Rotheneichner et al., 2018; Benedetti et al., 2020). Cells in the dorsal and ventral striatum (dSt and vSt: Bregma 1.5, 1.1, 0.7 mm) were quantified in the same way as the ones in the Pir, and cell density was calculated as number of cells per mm^2^ of the ROI. In the olfactory tubercle (OT: Bregma 1.7–1.1 mm) due to the low density of cells, counts were performed with a manual counter at higher magnification, and cell density is presented in number of cells per coronal section. Similarly, in the DG (Bregma –1.46, –2.2 mm), cells were quantified with a manual counter and corrected by the perimeter of the structure. In the OB, due to the high density of cells and fibres, we split the image in the RGB channels, selected the red channel and calculated the OD of the inverted image as Log10 = Max Intensity/Mean Intensity.

The presence of reelin-expressing cells was similarly analysed according to the stereotaxic atlas at the following structures: OB, mitral and periglomerular (PGL) layers; OT; Pir, layers 1, 2, and 3; and DG, at both granular and polymorph layers (GrDG and PoDG, respectively). Microphotographs were obtained using a digital camera (Olympus XC50) associated with an Olympus CX41RF-5 optical microscope. The reelin-expressing cells within the OT, hippocampus and layers 1 and 3 of Pir were counted manually with the aid of the multi-point tool of ImageJ, whereas the OB and Pir’s layer 2 were evaluated based on the levels OD and fraction of the area occupied.

### Statistical analysis

Data were analysed using the software IBM SPSS Statistics 22.0. We first removed extreme values (Tukey’s hinges method, values lying farther than three interquartile range in a boxplot) and checked normality and homocedasticity of the data. Then, behavioural and histological data were analysed with two-way analysis of variance (ANOVA) with group (naïve or MS) and genotype (WT or *Mecp2*-null) as between-subject sources of variance, followed by *post hoc* Bonferroni correction for multiple tests. Significance was set at *p* < 0.05 for individual factors, and we carried out *post hoc* comparisons when *p* < 0.1 for the interaction between factors.

Moreover, to have a multivariate view of the problem, a Principal Component Analysis (PCA) was performed using the “factoextra” package (Kassambara and Mundt, 2020) of the R language (R Development Core Team, 2019; R Core Team, 2020).

## Results

WT and *Mecp2-*null adolescent males (age 5–6 weeks old), naive and MS groups, were submitted to a battery of behavioural tests starting from the less to the more aversive conditions, one per day: EPM under red light, OF and FST. The following week, mice were tested in the second EPM under bright light conditions, 1 h after which animals were sacrificed to assess c-FOS activation patterns and neuronal markers.

### Maternal separation increases the weight of wild-type adolescent mice

**FIGURE 2:**
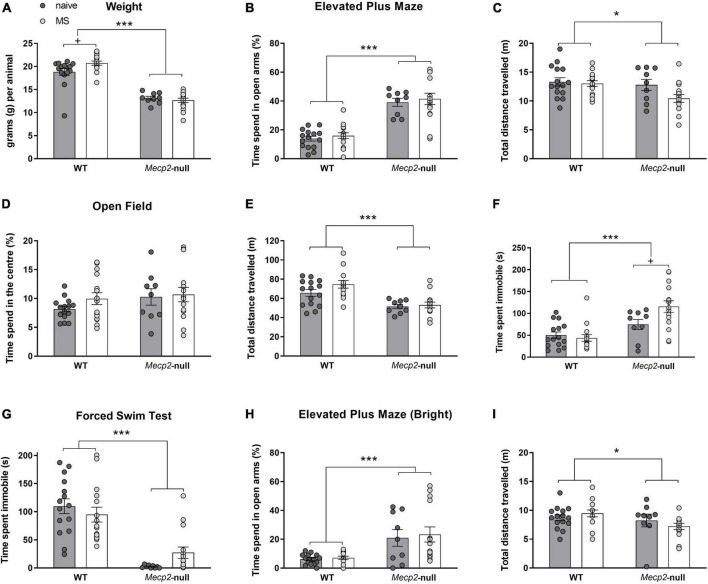
Adolescent *Mecp2*-null males show motor deficits and reduced anxiety- and depressive-like behavioural responses. Males from both genotypes, WT and *Mecp2*-null, from either MS or naïve conditions were weighted and tested sequentially for EPM, OF and FST (WT-naïve, *n* = 15; WT-MS, *n* = 15, *Mecp2*-null-naïve, *n* = 9; *Mecp2*-null-MS, *n* = 15). **(A)** Body weight of the animals. In the EPM, **(B)** percentage of time in the open arms of the maze, and **(C)** distance travelled. In the OF, **(D)** percentage of time spent in the centre of the arena, and **(E)** total distance travelled. Time spent immobile **(F)**. In the FST, **(G)** mutant mice spend significantly less time immobile than their WT counterparts. One week after previous behavioural assays, WT and *Mecp2*-null male mice, from either MS or naïve conditions were re-exposed to EPM under bright light conditions. **(H)** Time in the open arms of the maze and **(I)** Distance travelled. Graph show individual values and mean ± standard error mean (SEM) for each group. *p < 0.05, ***p < 0.001 by genotype; ^+^*p* < 0.05 by group. EPM, elevated plus maze; FST, forced swimming test; MS, maternal separation; OF, open field; WT, wild-type.

Before starting the behavioural battery, the body weight of all animals was registered. The ANOVA revealed a significant main effect of the factor genotype (*F*_1,50_ = 137.009, *p* < 0.001, η^2^ = 0.733) and a significant interaction between genotype and group (*F*_1,50_ = 4.140, *p* = 0.047, η^2^ = 0.076). Subsequent pairwise comparisons with the Bonferroni correction revealed that WT-MS males were significantly heavier than their WT-naïve counterparts (*p* = 0.017, η^2^ = 0.108), but no effects were found in *Mecp2*-null animals (*p* > 0.5; Figure 2A).

### *Mecp2*-null mice display decreased locomotor and anxiety-like phenotype in the elevated plus maze, and maternal separation increases their immobility in the open field

#### Elevated plus maze

Animals were first tested in the EPM (under red light conditions) in a 5-min session. Regarding the anxiety-like responses, the ANOVA revealed no effect of group (*F*_1,50_ = 0.576, *p* = 0.451) or interaction (*F*_1,50_ = 0.004, *p* = 0.950) in the percentage of time spent by mice in the open arms of the maze. Nonetheless, we found a strong effect of genotype (*F*_1,50_ = 77.675, *p* < 0.0001, η^2^ = 0.608), showing a decreased anxiety-like phenotype in *Mecp2*-null males (Figure 2B). The ANOVA for total distance travelled revealed a significant effect of the factor genotype (*F*_1,50_ = 31.466, *p* = 0.032, η^2^ = 0.089) and a marginal effect of group (*F*_1,50_ = 3.439, *p* = 0.070, η^2^ = 0.064), with no significant interaction between factors (genotype × group, *F*_1,50_ = 2.086, *p* = 0.155), with *Mecp2*-null animals travelling a smaller total distance (Figure 2C). To determine whether the decrease in anxiety like behaviour in *Mecp2*-null mice persist regardless of their decrease in motility, we also analysed the number of entries to the open arms after normalising by distance travelled (WT-naïve, 0.42 ± 0.04; *Mecp2*-null-naïve 0.85 ± 0.05; WT-MS 0.44 ± 0.06, *Mecp2*-null-MS 0.86 ± 0.03). Similarly, this analysis revealed a strong significant effect of genotype (*F*_1,50_ = 77.97, *p* < 0.001, η^2^ = 0.609) with no effect of group (*F*_1,50_ = 0.057, *p* = 0.811) or the interaction (*F*_1,50_ = 0.008, *p* = 0.929). Thus, as expected, both anxiety-like phenotype and motor parameters were significantly affected by genotype.

#### Open field

Spontaneous locomotor activity and exploration were measured in the OF apparatus in a 20-min session. Regarding time spent in the centre of the apparatus in relation to the remaining area, we did not find a statistically significant effect of any factor (*p* > 0.1; Figure 2D).

The analysis of the total distance travelled in the maze showed a statistically significant effect of genotype (*F*_1,50_ = 25.733, *p* < 0.001, η^2^ = 0.340; Figure 2E) with *Mecp2*-null males travelling a smaller total distance than their WT controls. Interestingly, the analysis of total immobility revealed both a significant effect of genotype (*F*_1,50_ = 21.626, *p* < 0.0001, η^2^ = 0.302), with significantly higher immobility in *Mecp2*-null mice, and of the interaction of genotype and group (*F*_1,50_ = 5.206, *p* = 0.027, η^2^ = 0.094; Figure 2F). *Post hoc* pairwise comparisons revealed that MS significantly increased the immobility in *Mecp2*-null mice (*p* = 0.012, η^2^ = 0.120), but not in the WT mice (*p* = 0.640).

### Forced swimming test

The performance of WT and *Mecp2*-null males was evaluated in the FST by measuring the immobility time in a 5-min session. Two-way ANOVA revealed a strong significant effect of genotype in the total time of immobility (*F*_1,50_ = 52.342, *p* < 0.001, η^2^ = 0.511; Figure 2G) and no significant effects of either group or the interaction between factors (*p* > 0.1). However, a closer inspection of the distribution of the data on immobility in the *Mecp2*-null groups (Figure 2G) revealed that time spent immobile by *Mecp2*-null animals of the MS group was ∼10-fold increased (11.53 ± 4.25) with respect to the average of their naïve counterparts, whereas this effect was not seen in the WT group (0.86 ± 0.12).

### Elevated plus maze-bright

Finally, all animals were tested in a more anxiogenic version of the EPM, under bright light conditions. Again, the percentage of time spent in the open arms of the maze was strongly affected by genotype (*F*_1,49_ = 16.461, *p* < 0.001; Figure 2H), with *Mecp2*-null mice spending more time in the open arms. In addition, we observed a significant effect of genotype in the total distance travelled among groups (*F*_1,49_ = 4.964, *p* = 0.03, η^2^ = 0.09; Figure 2I). As with previous EPM in standard conditions, we also analysed the number of entries to the open arms after normalising by distance travelled (WT-naïve, 0.30 ± 0.04; *Mecp2*-null-naïve 0.55 ± 0.12; WT-MS 0.31 ± 0.05, *Mecp2*-null-MS 0.58 ± 0.08). Again, this analysis showed that genotype was highly significant (*F*_1,49_ = 14.34, *p* < 0.001, η^2^ = 0.226) while neither group (*F*_1,49_ = 0.060, *p* = 0.807) nor the interaction (*F*_1,49_ = 0.0205, *p* = 0.887) were statistically significant.

### Maternal separation and lack of *Mecp2* affect neuronal activation patterns upon exposure to the elevated plus maze

**FIGURE 3:**
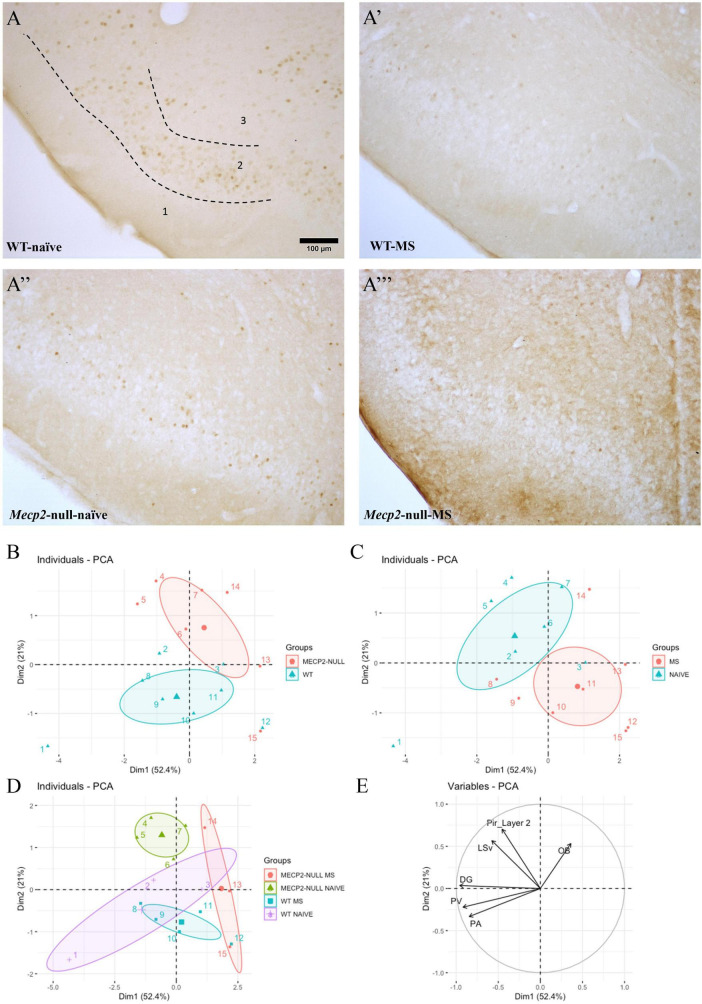
Exposure to the EPM in bright conditions leads to differential c-FOS activation at multiple brain areas that is genotype- and group-dependent. One hour after the re-exposure to the EPM behavioural test under bright conditions, a batch of animals from each genotype x treatment were culled to assess c-FOS immunoreactivity (c-FOS-ir; *n* = 3-6, depending on genotype/treatment/area). Representative photomicrographs from c-FOS immunostaining at the Pir from **(A)** WT-naïve, **(A’)** WT-MS, **(A”)** *Mecp2*-null-naïve, **(A”’)** *Mecp2*-null-MS. Scale bar represents 100 μm. Principal component analysis of c-FOS expression profiles at the different areas studied are coloured according to **(B)** genotype, **(C)** group or **(D)** both; **(E)** arrows displaying the weight of the variables (brain areas) for both first and second principal components. Graphs show individual values and mean for each group. DG, dentate gyrus of the hippocampus; OB, olfactory bulb; PA, paraventricular hypothalamic nucleus; PV, paraventricular thalamic nucleus; Pir, piriform cortex; LSV, ventral part of the lateral septum; EPM, elevated plus maize; MS, maternal separation; WT, wild-type.

PCA of the c-FOS positive nuclei (Figures 3A–A”’) was performed considering the different areas analysed as variables. Using first and second components PCA is able to explain more than 70% of the variability (52 and 21%, respectively). When plotted, these two components show data clustering according to genotype (WT vs. *Mecp2*-null) and group (Naïve vs. MS). On the one hand, “genotype” spreads in the bisector 2nd quadrant to 3rd quadrant with the variable OB determining the direction of the *Mecp2*-null while the combinatorial effect of PV and Pa variables determines the directionality of WT. On the other hand, “group” spreads between 1st and 4th quadrants and the difference seems to be defined by LSV and Pir layer 2. If assessing the classification in terms of genotype and group, there is a closer distance between groups as MS from both genotypes are clustered in the 4th quadrant, while *Mecp2*-null-naïve are in the 1st quadrant and WT-naïve in the 3rd. Nonetheless, the condition WT-naïve shows a greater degree of variability probably due to the low *n* number (Figures 3B–E).

**FIGURE 4:**
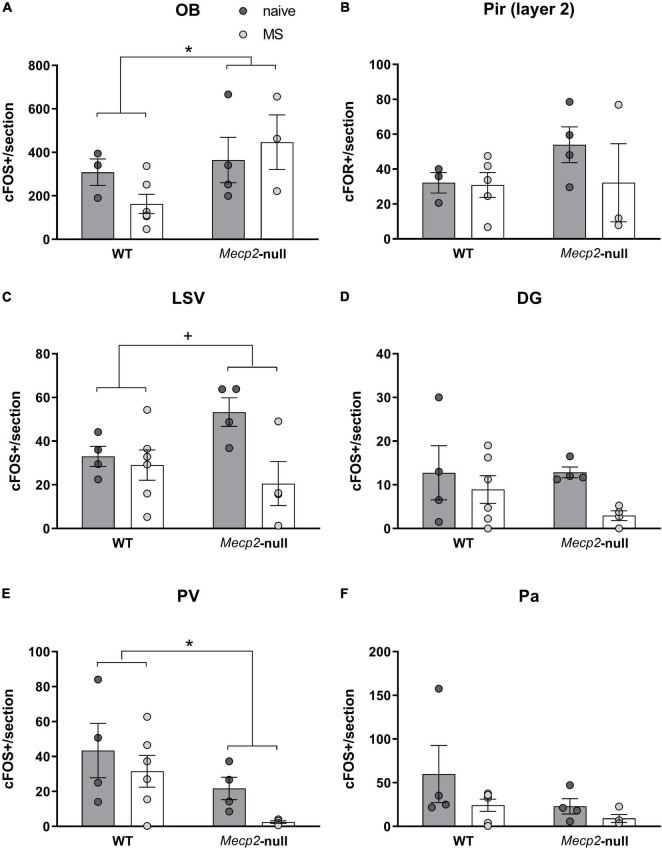
c-FOS-ir at multiple brain areas is dependent on Mecp2 dosage and MS. Number of c-FOS positive cells per section at the **(A)** OB, **(B)** layer 2 of the Pir, **(C)** LSV, **(D)** DG of the hippocampus, **(E)** PV, and **(F)** Pa. All graphics describe individual values and mean ± SEM. *p < 0.05 by genotype; ^+^*p* < 0.05 by group. DG, dentate gyrus of the hippocampus; OB, olfactory bulb; PA, paraventricular hypothalamic nucleus; PV, paraventricular thalamic nucleus; Pir, piriform cortex; LSV, ventral part of the lateral septum.

We also analysed the level of c-FOS expression in each of the structures by means of an ANOVA. We found a significant modest effect only of the factor genotype in the OB (*F*_1,11_ = 5.111, *p* = 0.045, η^2^ = 0.317), with *Mecp2*-null mice expressing higher levels of c-FOS in this structure (Figure 4A). The higher levels of c-FOS were not reflected in the Pir, where we did not find significant effects of either factor or their interaction (all *p* > 0.3) (Figure 4B). In LSV, by contrast, we found a significant effect of group (*F*_1,14_ = 5.996, *p* = 0.028, η^2^ = 0.300), but not genotype (*F*_1,14_ = 0.614, *p* = 0.446), and a trend in their interaction (*F*_1,14_ = 3.674, *p* = 0.076; η^2^ = 0.208) (Figure 4C). *Post hoc* comparisons with the Bonferroni correction revealed that c-FOS in this area was significantly decreased by MS in the *Mecp2*-null males only (*p* = 0.010). We did not find significant effects of either factor or their interaction in the DG (all *p* > 0.1) (Figure 4D).

Finally, in the PV, we found a significant effect of genotype (*F*_1,14_ = 6.686, *p* = 0.022; η^2^ = 0.323), but not group or interaction between factors (*p* > 0.1), this time with lack of *Mecp2* leading to a decrease in c-FOS expression (Figure 4E), whereas we did not find significant effects of either factor or their interaction in the Pa (all *p* > 0.1) (Figure 4F).

### Maternal separation and *Mecp2* deficiency increase the density of doublecortin immature cells in a region-specific manner

**FIGURE 5:**
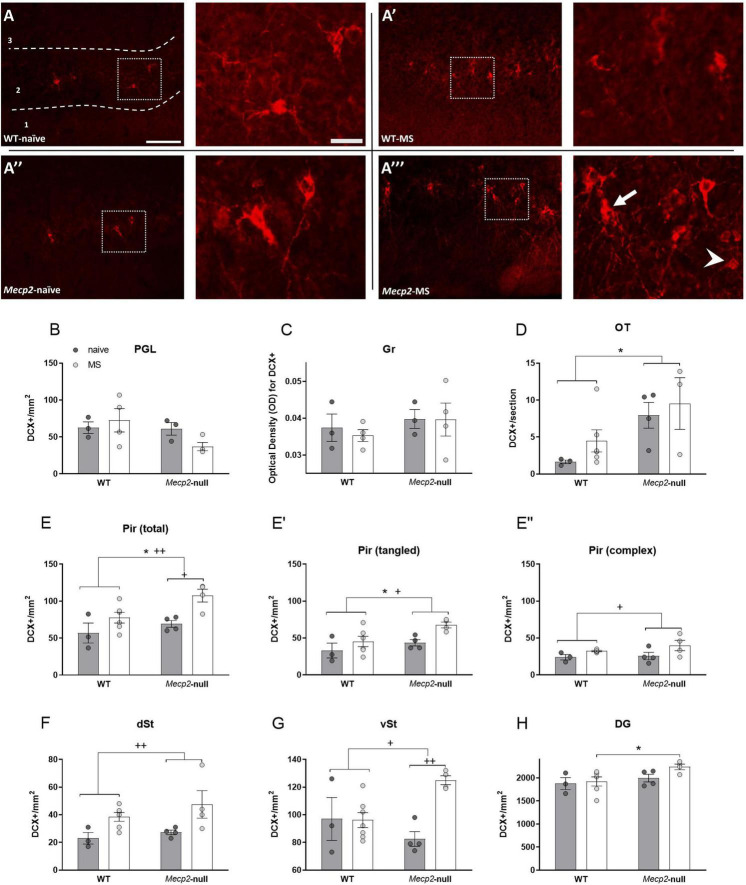
Lack of *Mecp2* increases the number of DCX positive cells in a region-specific manner and MS further exacerbates this effect. **(A)** Representative photomicrographs from DCX immunostaining (red) at the Pir from WT-naïve **(A)**, WT-naïve, WT-MS **(A’),** *Mecp2*-null-naïve **(A”)** and *Mecp2*-null-MS at 10× (left panel) (scale bar 100 μm) and a higher magnification at 20× (right panel) (scale bar 25 μm) Arrow points to a complex and arrowhead to a DCX-ir tangled representative DCX-ir neurons. In the OB: density of DCX-ir cells at the PGL **(B)** and OD for DCX at the Gr **(C)**. **(D)** DCX-ir cells in OT and **(E)**. total density of DCX-ir in the Pir, and in the tangled **(E’)** and complex DCX-ir cells only **(E”)**. Number of DCX positive cells at **(F)** dSt and **(G)** vSt. Number of DCX-ir cells in the DG when comparing animals from the MS groups **(H)**. All graphs show individual values and mean ± SEM. *p < 0.05 by genotype; ^+^*p* < 0.05, ^++^*p* < 0.01 by group. DCX, doublecortin; DG, dentate gyrus of the hippocampus; dSt, dorsal striatum; Gr, granular layer of the olfactory bulb; OB, olfactory bulb; OT, olfactory tubercle; PGL, periglomerular layer of the olfactory bulb; Pir, piriform cortex; vSt, ventral striatum.

In our previous study in *Mecp2*-null males we discovered an excess of DCX-ir immature neurons specifically in the Pir (Figures 5A–A”’) and the OT, with no effect in other neurogenic areas (Martínez-Rodríguez et al., 2019). In agreement with this study, we did not find significant effects of either group or genotype in the OB as assessed at both PGL and Gr (all *p* > 0.1, Figures 5B,C), but we found a significant effect of genotype in the OT (Figure 5C; *F*_1,14_ = 8.081, *p* = 0.015, η^2^ = 0.402), with *Mecp2*-null males displaying increased density of DCX-ir cells (Figure 5D). Also, the ANOVA revealed the expected significant effect of genotype in the total number of DCX-ir cells in the Pir (*F*_1,14_ = 6.059, *p* = 0.029; η^2^ = 0.318), accompanied by a significant effect of group (*F*_1,14_ = 11.921, *p* = 0.004, η^2^ = 0.478; Figure 5E). DCX-ir cells in the Pir have been described as tangled (more immature) and complex (in transition to maturity) (Rotheneichner et al., 2018) (Figure 5A”’). Thus, we analysed these two types separately and found that tangled cells were increased by both *Mecp2* deficiency (*F*_1,14_ = 5.691, *p* = 0.033, η^2^ = 0.304) and MS (*F*_1,14_ = 6.956, *p* = 0.02, η^2^ = 0.020; Figure 5E’), whereas for complex cells we found only an effect of group (*F*_1,14_ = 6.855, *p* = 0.021, η^2^ = 0.345; Figure 5E”). Thus, both lack of MeCP2 and MS increases the density of DCX-ir in this cortical area, with genotype affecting specifically to tangled, more immature forms.

In the dST, next to dorsal tip of the lateral ventricles, we found a significant effect of group (*F*_1,14_ = 11.215, *p* = 0.05, η^2^ = 0.445; Figure 5F) whereas in the vST, next to the ventral tip of the lateral ventricles, we found a significant interaction of group x genotype (*F*_1,14_ = 8.779, *p* = 0.01, η^2^ = 0.385; Figure 5G). *Post hoc* test with Bonferroni correction revealed that MS increased the number of DCX-ir in this latter area specifically in the *Mecp2*-null animals (*p* = 0.008, η^2^ = 0.541). Finally, in the DG, we found an effect of genotype (*F*_1,14_ = 4.846, *p* = 0.046, η^2^ = 0.272), not reported previously, and a trend toward interaction of genotype x group (*F*_1,14_ = 3.486, *p* = 0.085, η^2^ = 0.211). The *post hoc* test with Bonferroni correction revealed that the density of DCX-ir cells was significantly increased in the *Mecp2*-null-MS group as compared with WT-MS (*p* = 0.008, η^2^ = 0.433; Figure 5H), but not in the *Mecp2*-null-naïve compared to WT-naive.

Taken together, our results confirm and extend our previous results about the effect of lack of MeCP2 in increasing DCX-ir in a region-specific manner, and further reveals that MS can cause a similar effect in WT mice, while exacerbating the alteration in *Mecp2*-null animals.

### Both maternal separation and lack of *Mecp2* reduce the expression of reelin

**FIGURE 6:**
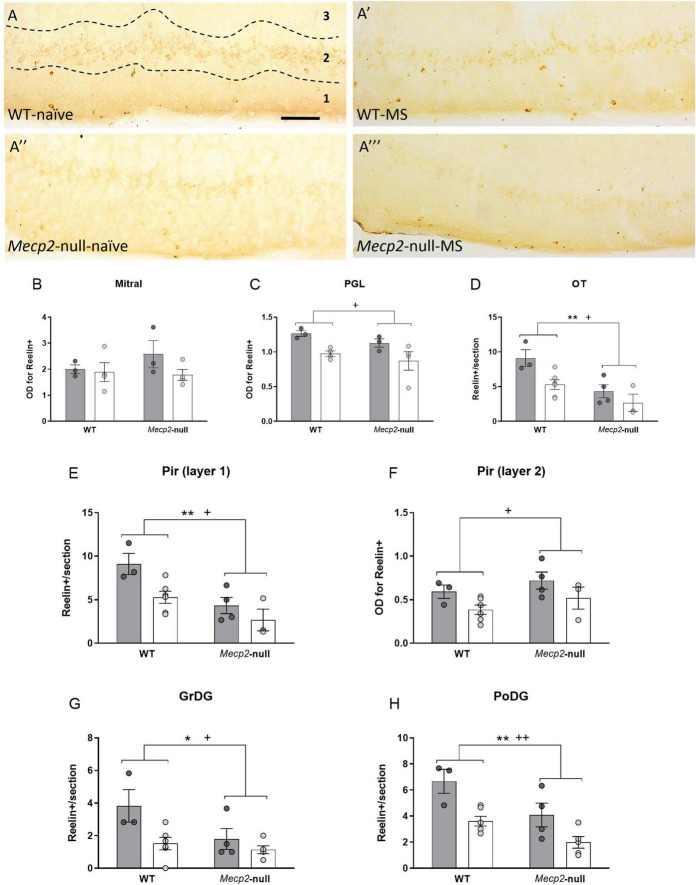
Both MS and the lack of *Mecp2* reduce the expression of reelin. **(A)** Representative photomicrographs from reelin immunostaining (dark-brown, revealed with DAB) at the Pir from WT-naïve **(A)**, WT-MS **(A’)**, *Mecp2*-null-naïve **(A”)** and *Mecp2*-null-MS **(A”’)**; scale bar represents 100 μm. Note that while the positive signal in the Pir layer 1 is restricted to a population of cellular bodies, the distribution in Pir layer 2 is diffuse. Reelin-ir in the OB **(B)** and the PGL **(C)**. Number of reelin immunopositive cells per section at the OT **(D)** and layer 1 of the Pir **(E)** and reelin-ir in layer 2 of the Pir **(F)**. Reelin-ir cells in the **(G)** GrDG and **(H)** PoDG. In all cases, graphs represent individual values and mean ± SEM. *p < 0.05, **p < 0.01 by genotype; ^+^*p* < 0.05, ^++^*p* < 0.01 by group. GrDG, granular cell layer of the dentate gyrus of the hippocampus, OT, olfactory tubercle; PGL, periglomerular layer of the olfactory bulb; Pir, piriform cortex; PoDG, polymorph cell layer of the dentate gyrus of the hippocampus.

Reelin immunostaining revealed the presence of discrete neurons in the layer 1 of the OT and Pir (Figures 6A–A”’), and in the DG of the hippocampus. By contrast, immunoreactivity in the PGL and mitral layers of the OB, and layer 2 of Pir was diffuse and did not allow for direct quantification of single cells (Figures 6A–A”’). Thus, we quantified the density of reelin-ir cells in the first group of brain areas, and optic density in the latter. In the OB, ANOVA revealed no significant effect of either genotype, group or their interaction (all *p* > 0.200) in the mitral cell layer, but we found a significant effect of group (*F*_1,14_ = 9.744, *p* = 0.011, η^2^ = 0.494) in the PGL, suggesting a reduction of reelin-ir cells induced by MS in both genotypes (Figures 6B,C). In the OT, we found a significant effect of genotype (*F*_1,14_ = 8.092, *p* = 0.015, η^2^ = 0.403), with *Mecp2*-null males having less reelin-ir cells as compared to WT (Figure 6D). In layer 1 of the Pir, we found significant effects of both genotype (*F*_1,14_ = 14.121, *p* = 0.003, η^2^ = 0.541) and group (*F*_1,14_ = 7.755, *p* = 0.017, η^2^ = 0.393), again with both factors reducing the number of reelin-ir cells (Figure 6E). In layer 2 of the Pir, by contrast, only MS reduced the expression of reelin (group, *F*_1,14_ = 5.534, *p* = 0.037, η^2^ = 0.316; Figure 6F). Finally, in the hippocampus, we found significant effects of both genotype and group in both the GrDG (genotype, *F*_1,14_ = 5.260, *p* = 0.038, η^2^ = 0.273; group, *F*_1,18_ = 7.950, *p* = 0.014, η^2^ = 0.362; Figure 6G) and PoDG (genotype, *F*_1,14_ = 11.186, *p* = 0.005, η^2^ = 0.444; group, *F*_1,14_ = 16.767, *p* = 0.001, η^2^ = 0.545; Figure 6H). Thus, whereas in the case of DCX-ir either lack of MeCP2 and/or MS caused an increase in this marker, both factors caused a decrease in the expression of reelin, with either factor having a specific weight in a region-dependent manner.

## Discussion

In this study, we used *Mecp2-*null male mice and their WT siblings to assess the interaction between lack of MeCP2 and an early life stressor (i.e. MS) in behaviour and in the maturation and functionality of underlying neuronal circuits in adolescence. We found that MS leads to an increase in body weight and to a region-specific increase in the number of DCX-ir immature neurons in adolescent WT males. Further, compared to WT littermates, adolescent *Mecp2*-null males showed hypolocomotion and a reduction in both anxiety and depressive-like behaviours, with MS exacerbating immobility, without apparent impact on the emotional component. At the histological level, both MS and lack of *Mecp2* increased the density of DCX neurons and decreased the density of reelin neurons.

### Effect of *Mecp2* deficiency and maternal separation in anxiety-like responses and neuronal activation

At 6 weeks of age, *Mecp2*-null male mice already show behavioural signs of motor impairments as observed in both the EPM and the OF tests, where *Mecp2*-null mice travel less distance regardless of the exposure to MS. These findings are in line with previous publications, where the hemizygosis condition of the males leads to a pathological phenotype associated with RTT at earlier ages than in heterozygous females (Guy et al., 2001; Ribeiro and MacDonald, 2020). Also, comparable to *Mecp2* heterozygous females (Abellán-Álvaro et al., 2021), *Mecp2*-null male mice show a significant decrease in anxiety-like behaviours specifically in the EPM, but not the OF. Additionally, FST revealed that *Mecp2*-null males have lower immobility than WT, which could suggest an antidepressant-like phenotype associated with the lack of *Mecp2* similar to that we reported in females (Abellán-Álvaro et al., 2021). Alternative interpretations to the fact that *Mecp2*-null males are more mobile in the FST might include an increased hypothermia due to lower body weight or that this response is associated with increased reactivity to a stressful situation, that is, an active coping strategy (Armario, 2021). This active coping strategy in form of increased mobility might not be able to show in “land” assays because at the age tested males are already symptomatic, or simply the OF and EPM tests are not appropriate to discriminate an active or passive coping strategy (Armario, 2021).

In agreement with sensory-motor impairments acting as confounding factors, a recent study found that *Mecp2-*het females with trimmed whiskers did not show the anxiolytic phenotype in the EPM, so authors suggested that preference for the open arms of *Mecp2-*het females in the EPM might reflect sensory hypersensitivity (Flores Gutiérrez et al., 2020). Nevertheless, we previously found that c-FOS expression in the CRH cells of the Pa of *Mecp2*-het females was decreased after EPM as compared to WT littermates (Abellán-Álvaro et al., 2021), supporting decreased physiological stress response. In summary, aberrant behaviours in *Mecp2*-deficient models might be the result of the interaction between sensory-motor impairments and altered circuit functionality.

There also seems to be an interaction between MS and the dosage of functional *Mecp2* in terms of behaviour. *Mecp2*-null animals that were previously exposed to MS spend more time immobile in the OF test than their naïve counterparts, whereas MS did not affect this parameter in their WT siblings. Immobility in this behavioural assay can be regarded as either exacerbated motor impairments or the result of freezing, which is associated with increased anxiety (Lezak et al., 2017). Therefore, the interpretation of this correlation must be done with caution as could be: (a) MS is exacerbating the motor deficits associated with this loss-of-function mutation or (b) lack of *Mecp2* increases the susceptibility of MS to enhance anxiety-like responses. Nonetheless, given the decrease in anxiety-like behaviour observed in the EPM, and that *Mecp2*-het females from the same line are described with less anxious characteristics (Abellán-Álvaro et al., 2021), it is more likely that ELS is exacerbating the motor deficits.

### Neuronal activation pattern in anxiety/stress-related brain regions

The c-FOS data analysis by both PCA and ANOVA showed that some cerebral areas are differentially activated depending on the genotype while others are more relevant to the exposure to an early life stressor. The lack of *Mecp2* seems to be associated with an increased activation at the OB and a reduction at the PV in adolescent male mice. The increased activation at the OB does not lead to a similar increase on the activity of Pir, where this information gets integrated (Martínez-García et al., 2012). Thus, this peak of activity at the OB can be regarded as non-ethologically relevant and probably due to breathing disturbances, which have been previously observed in mutant male mice from one month of age onward (Viemari et al., 2005), a feature also present in RTT (Ramirez et al., 2013).

The diminished activity at the PV, however, bares a more difficult interpretation as this nucleus is important for the integration of multimodal signals including stress, emotional and feeding responses (Kirouac, 2015). The decrease of PV’s activity is not associated with a significantly diminished activity of the Pa, and thus we can presume the stress-response remains unaltered in the different genotypes and groups. Although this has not been assessed in the present study, a feasible association derived from the diminished activity of the PV observed in these *Mecp2*-null mice and unrelated to stress-reactivity, could be the altered visceral and neuropathic pain responses (Liang et al., 2020; Vigli et al., 2021; Irfan et al., 2022), which have been previously reported in heterozygous females (Samaco et al., 2013). Similarly, altered pain perception, including visceral, has been documented in RTT (Barney et al., 2015).

On the contrary, the LSV shows a more differential response to the EPM depending on the previous exposure to MS, especially in *Mecp2*-null males, whereas, as noted, we could not find significant differences in the Pa. Interestingly, in the female study, we observed significant changes in the Pa, whereas in the LSV we found only a similar trend towards hypo-activation associated with MS (Abellán-Álvaro et al., 2021). Differential effects between the female and the male study are also apparent in the behaviour in the bright EPM, since the anxiety-like phenotype was further diminished in both WT and *Mecp2*-het females by MS (Abellán-Álvaro et al., 2021), whereas EPM anxiety-like phenotype is only affected by genotype in the males. Sex and genotype-induced changes were also observed in their body weight, another phenotypical manifestation of RTT (Tarquinio et al., 2012). Mice lacking functional *Mecp2* are significantly smaller than WT siblings, which is in line with previous observations in young *Mecp2*-het females (data not shown). However, while MS did not affect body weight in the case of the females (data not shown), it seems to interact with the body weight of males. WT males exposed to MS are significantly heavier than WT controls, while MS does not induce a change in weight for the *Mecp2*-null mice. Thus, deficiency of *Mecp2* leads to smaller animals, potentially masking the increase in body weight associated with the exposure to an early life stressor. In summary, our data suggest that slightly different domains are susceptible to MS in males and females.

### Maternal separation exacerbates the increase in the density of doublecortin -ir neurons caused by lack of *Mecp2*, and both maternal separation and lack of *Mecp2* decrease reelin-ir in a region dependent manner

Immunoreactivity to DCX, a marker of immature neurons, shows that lack of *Mecp2* leads to an increase in the density of immature neurons at the Pir and the OT, similar to that we previously reported at early adulthood, 8 weeks of age (Martínez-Rodríguez et al., 2019). Further, MS uncovers an increase in the density of immature neurons at other regions, including the hippocampus, of adolescent *Mecp2*-null mice. Overall, our data suggests that the role of *Mecp2* in neuronal maturation is both region- and age-dependent in male mice, and susceptible to environmental conditions. Thus, MS seems to exacerbate the increased number of DCX cells at the Pir, suggesting that MS can interfere with neuronal maturation in a region-specific manner. Interestingly, the increase on DCX cells at the Pir is more explicit in the *Mecp2*-null mice. Therefore, lack of *Mecp2* seems to increase the susceptibility to an early life stressor in male mice in terms of neuronal maturation at the Pir, which could be associated with the intellectual disabilities reported in humans lacking functional *MECP2* (Orrico et al., 2000; Kudo et al., 2002).

Reelin, expressed during prenatal stages by Cajal-Retzius cells, is a secreted glycoprotein best known for its role during corticogenesis, maturation and migration of neurons (Tissir and Goffinet, 2003; Carceller et al., 2016). In the adult brain, it persists in a subset of cortical, cerebellar and hippocampal interneurons, among others with functions related to synaptic plasticity (Duveau et al., 2011). Notably, reelin expression is known to be under the epigenetic control of *Mecp2* (Sánchez-Lafuente et al., 2022). However, to our knowledge the expression of reelin has not been previously characterised in the *Mecp2*-null mice, with the exception of a report showing an increase of *Reln* transcript in the cerebellum (Jordan et al., 2007). By contrast, we report for the first time that lack of *Mecp2* leads to reduced reelin-ir neurons at the outermost layer of the Pir, the OT, and both the polymorph and the granular layers of the DG. Interestingly, exposure to MS *per se* resulted in a reduction in the expression of reelin in all the areas studied, regardless of the genotype of the mice. Therefore, early life stress in the form of MS could be reducing neuronal maturation/migration, in agreement to that previously reported at the hippocampus of various animal models of chronic stress (Brymer et al., 2018). Since the effect of MS on the decrease of reelin does not seem to interact with the lack of *Mecp2*, we suggest that MS might downregulate its expression in a pathway independent of the levels of *Mecp2*. Although not assessed in the present study, MS could be inducing DNA methylation at the promoter region of reelin, as it has been previously observed in other models of autism-like disorders and schizophrenia (Sánchez-Lafuente et al., 2022).

## Final remarks

Here, we assessed how the interaction between lack of *Mecp2* and ELS interfere with anxiety-like and depression-like behaviours as well as neuronal maturation in male mice. This study provides further evidence that deficiency of a functional *Mecp2* during early development may underlie an adolescent phenotype characterised by reduced anxiety- and depressive-like behaviours, together with some deficits in neuronal maturation in male mice.

Additionally, exposure to an early life stressor seems to have a long-lasting effect on some of these traits, and lack of *Mecp2* has the potential to exacerbate them further. In particular, the exposure to MS leads to increased immobility and increased density of immature neurons in the mutant animals, while precluding weight gain associated with this early life stressor.

Nonetheless, our research is not without limitation. This work aimed to complement our previous study using sibling female mice. However, direct comparison between sexes should be done with caution in this murine model since mutant males and females also differ in the dosage of functional *Mecp2*, which should also be accounted for. Additionally, another possible confounding factor could be the variance in the number of animals per cage and/or the reactivity states of the conspecifics, which can influence social behaviour in the critical period of adolescence (Endo et al., 2018; Kim et al., 2019) or the order in which the behavioural test was applied. Finally, although not feasible in the current experiment due to technical limitation, increasing the number of samples for the cellular comparisons would have helped strengthen our findings.

An increasing amount of evidence suggest that *Mecp2* has a role on the susceptibility to early life environmental disturbances and thus on determining the coping strategies that animals develop later in life (Abellán-Álvaro et al., 2021; Sánchez-Lafuente et al., 2022). Dosage-variation on functional *MECP2* during human neurodevelopment have been described in RTT, predominantly affecting females (Amir et al., 1999), but also affecting males in MDS (D’Mello, 2021; Pascual-Alonso et al., 2021) and in cases of somatic mosaicism and intellectual disability associated with RTT (Orrico et al., 2000; Kudo et al., 2002; Topçu et al., 2002; Venâncio et al., 2007). In the case of patients, these early life environmental disturbances are even more complicated to assess due to additional motor disabilities and communication impairments among other phenotypical manifestations. Therefore, these results reflect the complexity of assessing the causes of aberrant behaviours in both *Mecp2* mouse models and *MECP2*-associated disorders in humans, and emphasise that early life detrimental conditions could worsen altered phenotypes later in life, while reinforcing the importance of early intervention programs to a better disease prognosis.

## Data availability statement

The raw data supporting the conclusions of this article will be made available by the authors, without undue reservation.

## Ethics statement

The animal study was reviewed and approved by Comité de Ética y Experimentación Animal de la Universitat de València.

## Author contributions

JT-P performed research, analysed data, prepared figures, and wrote the manuscript. EM-R performed research and prepared figures. AF analysed the c-FOS data. CB-G performed research. OS provided resources and discussed results. EL participated in the design of the study and contributed to writing. MS designed the study, performed research, and was a major contributor in writing the manuscript and in funding acquisition. CA-P participated in the design of the study, supervised research and data analysis, and was a major contributor in writing the manuscript and in funding acquisition. All authors contributed to discuss results and read and approved the final manuscript.

## Abbreviations

ANOVA: analysis of variance
c-FOS: FOS proto-oncogene
DAB: 3-3’-diaminobenzidine
DCX: doublecortin
DG: dentate gyrus
dSt: dorsal striatum
ELS: early life stress
EPM: elevated plus maze
FST: forced swimming test
GrDG: granular layer of the DG
HPA: hypothalamic-pituitary-adrenal
LSV: lateral septum, ventral part
MDS: *MECP2* duplication syndrome
MeCP2: methyl-CpG binding protein 2
*Mecp2*-het: female mice heterozygous for a *Mecp2* loss-of-function mutation
*Mecp2*-null: male mice hemizygous for a *Mecp2* loss-of-function mutation
MS: maternal separation
NaBH_4_: sodium borohydride
NDS: normal donkey serum
NGS: normal goat serum
OB: olfactory bulbs
OD: optical density
OF: open field
OT: olfactory tubercle
Pa: Paraventricular hypothalamic nucleus
PB: phosphate buffer
PBS: phosphate buffered saline
PGL: periglomerular layer of the OB
Pir: piriform cortex
PND: postnatal day
PoDG: polymorph layer from the DG
PV: paraventricular thalamic nucleus
RT: room temperature
RTT: Rett syndrome
SEM: standard error mean
TBS: tris-buffered saline
Tx: Triton X100
vSt: ventral striatum
WT: wild-type.
